# Supplementary figures and images for: Prosapip1 (encoded by the Lzts3 gene) in the dorsal hippocampus mediates synaptic protein composition, long-term potentiation, and spatial memory
Source: eLife. 2025 Nov 26;13:RP100653. doi: 10.7554/eLife.100653 (PMC12656493; doi:10.7554/eLife.100653)

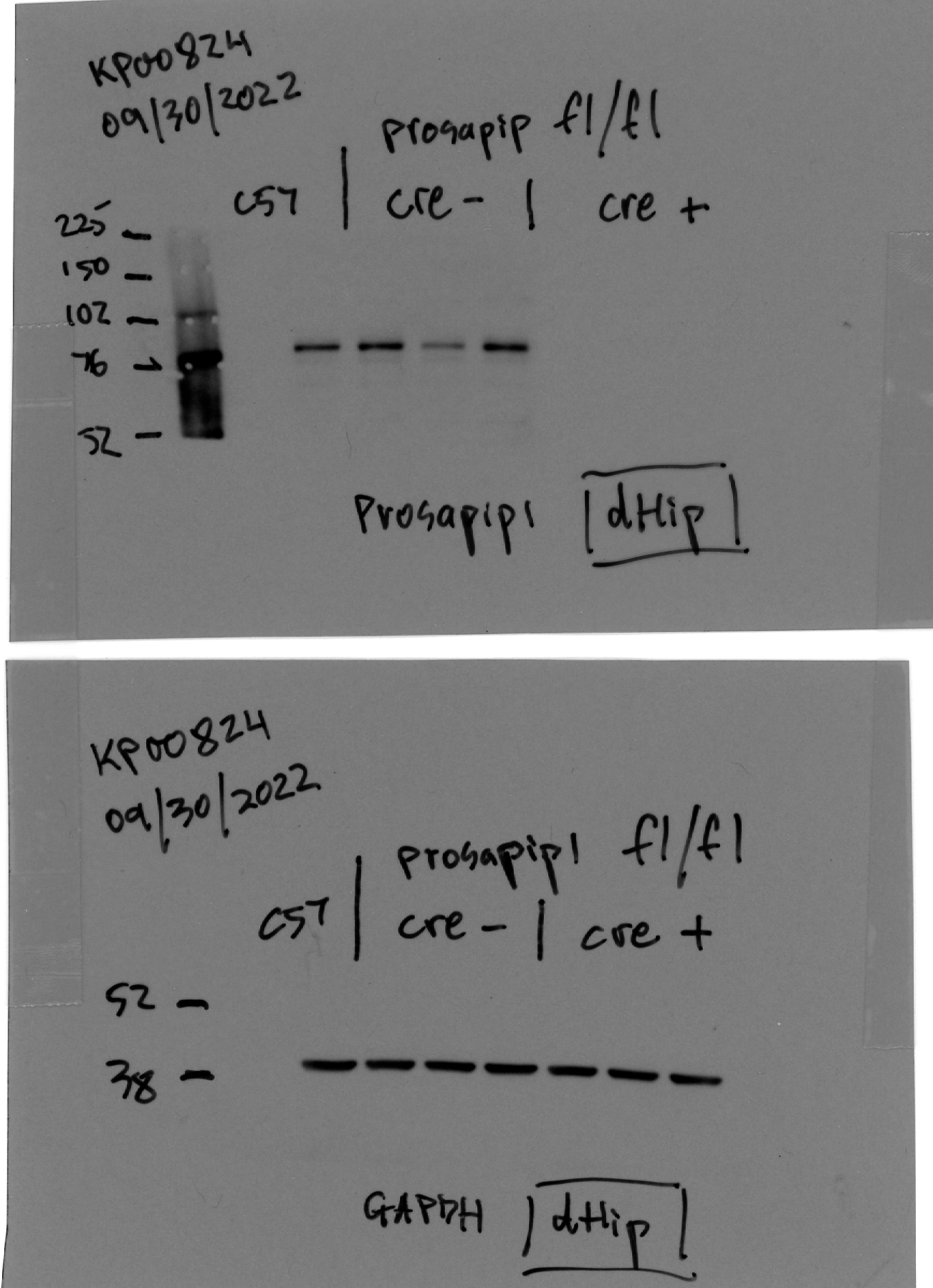

Supplement: Figure 1—source data 2. [file elife-100653-fig1-data2.zip › Figure 1 - Source Data 2/KP00824.tif]

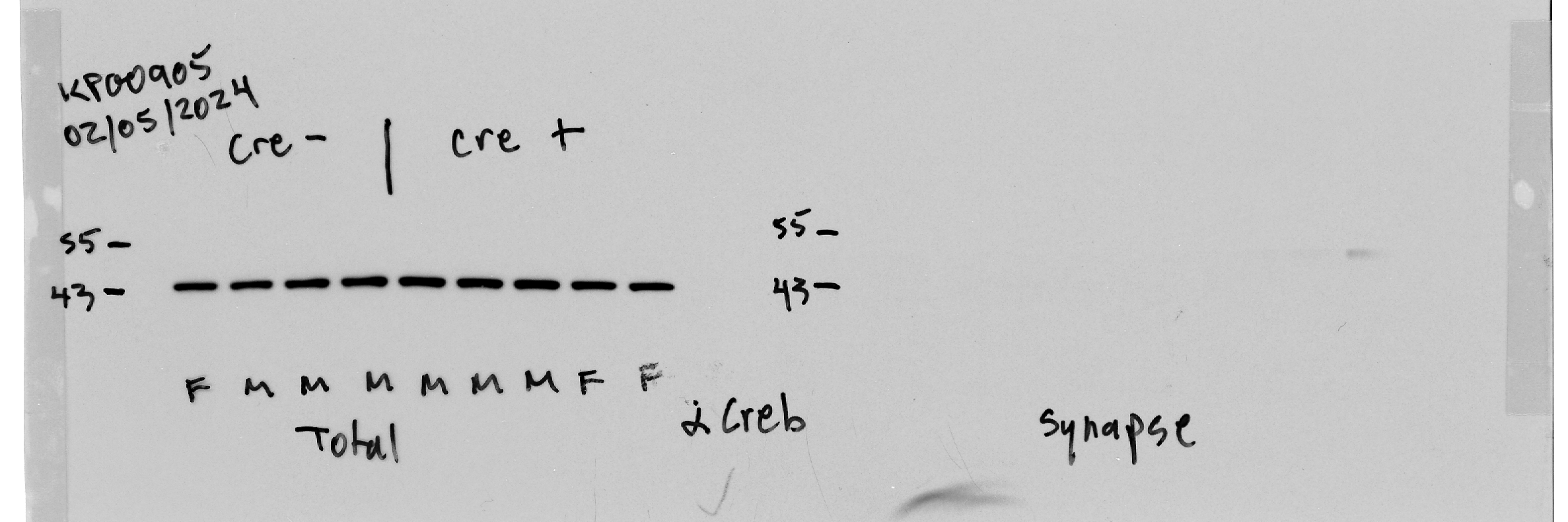

Supplement: Figure 2—source data 2. [file elife-100653-fig2-data2.zip › Figure 2 - Source Data 2/KP00905 Creb.tif]

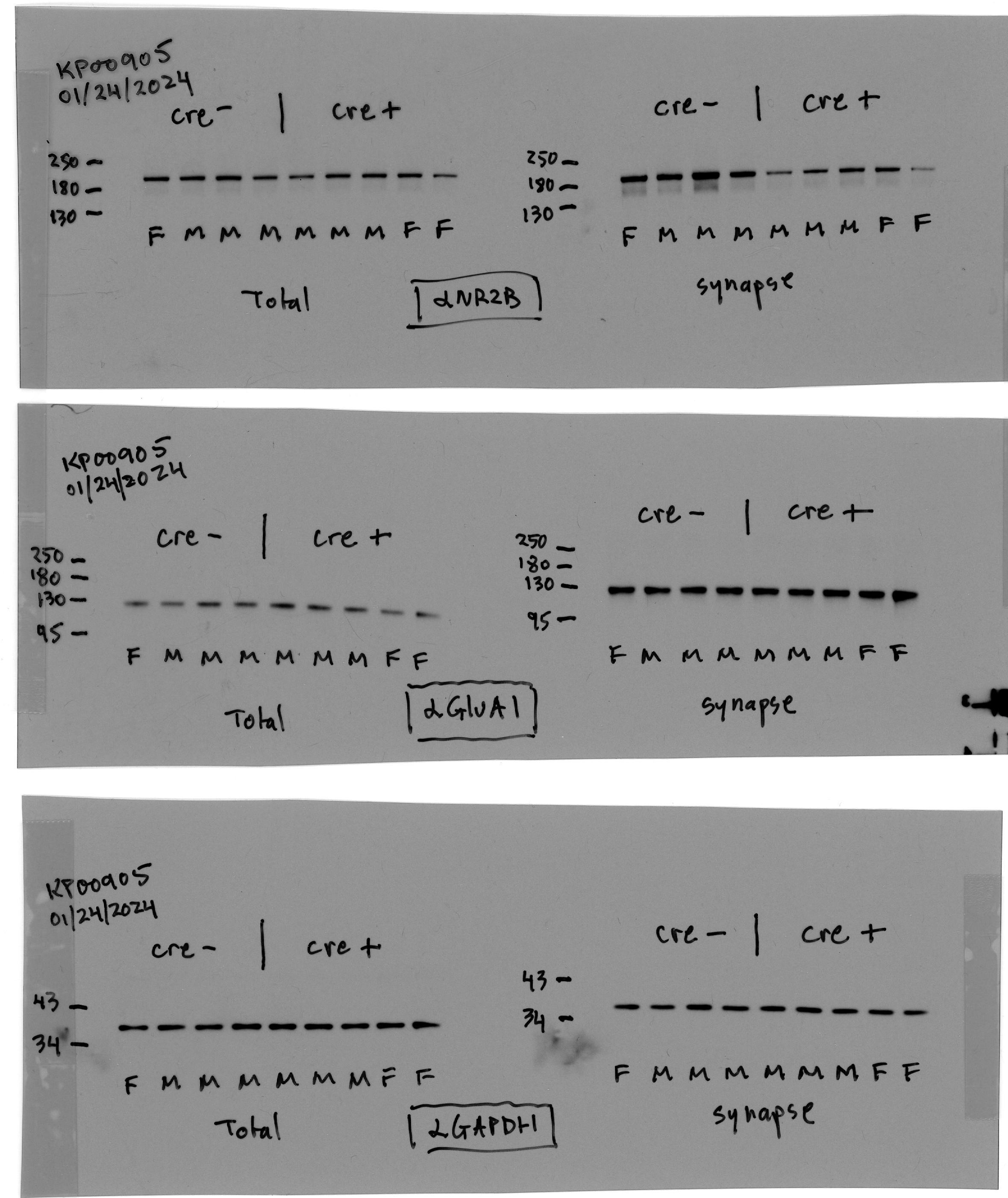

Supplement: Figure 2—source data 2. [file elife-100653-fig2-data2.zip › Figure 2 - Source Data 2/KP00905 NR2B GluA1 GAPDH.tif]

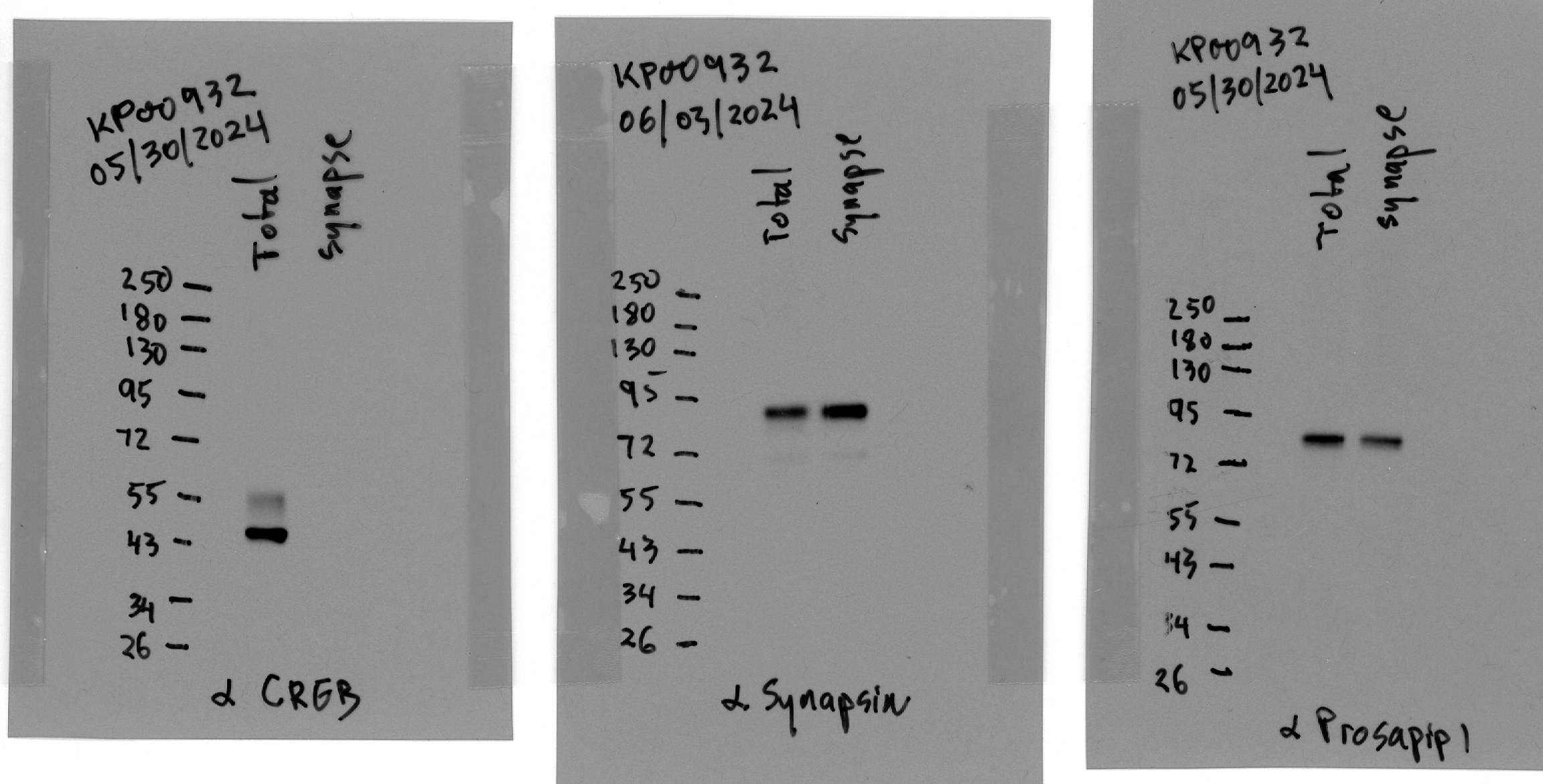

Supplement: Figure 2—source data 2. [file elife-100653-fig2-data2.zip › Figure 2 - Source Data 2/KP00932.tif]

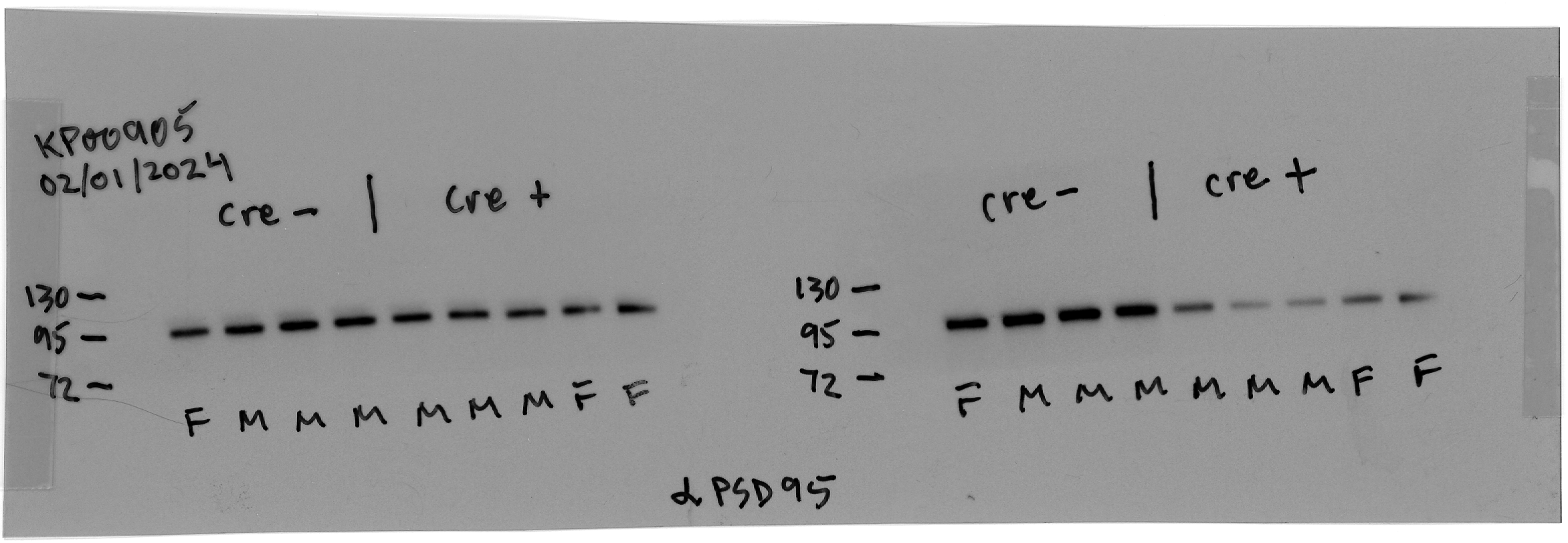

Supplement: Figure 2—source data 2. [file elife-100653-fig2-data2.zip › Figure 2 - Source Data 2/KP00905 PSD95.tif]

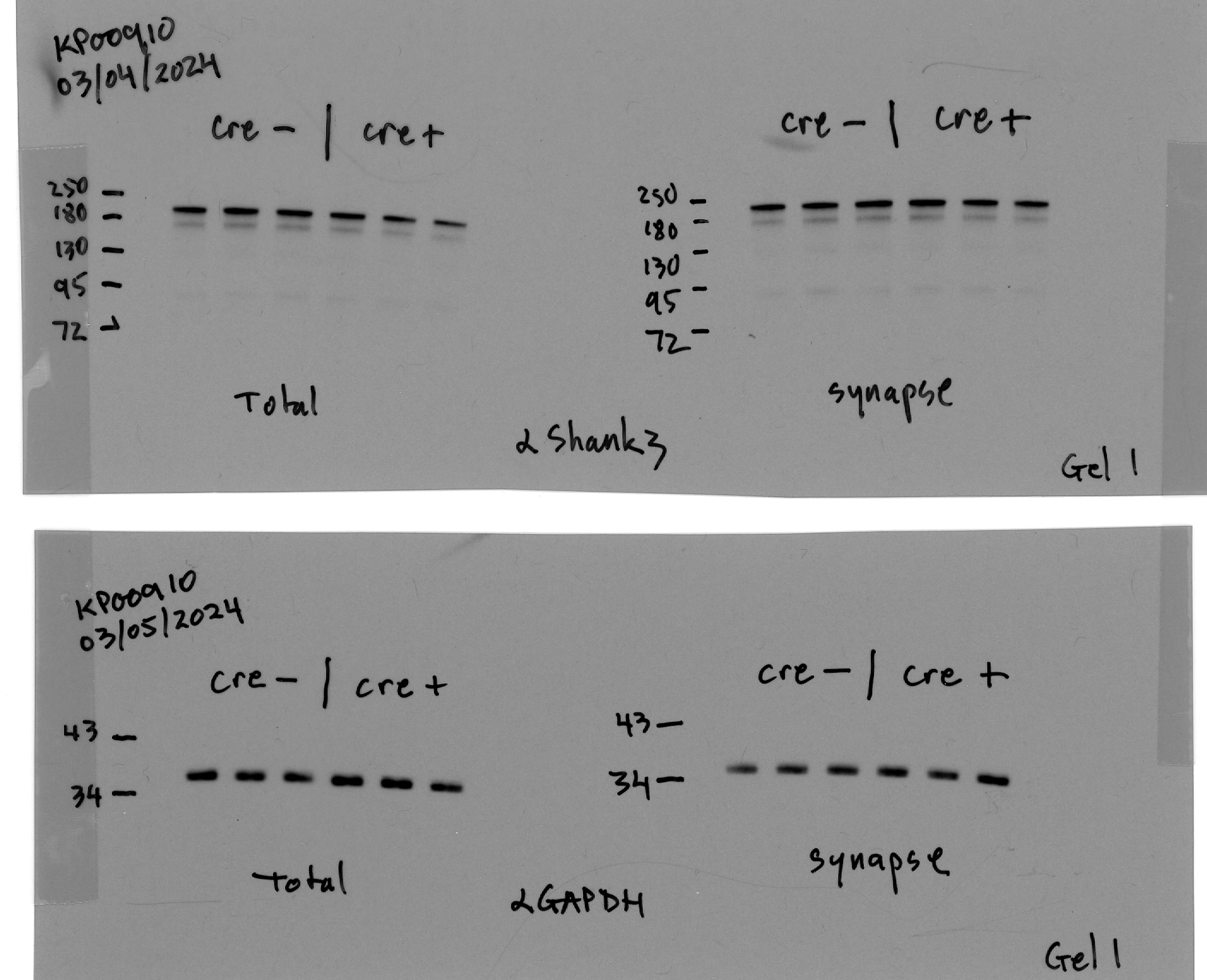

Supplement: Figure 2—source data 2. [file elife-100653-fig2-data2.zip › Figure 2 - Source Data 2/KP00910 gel1.tif]

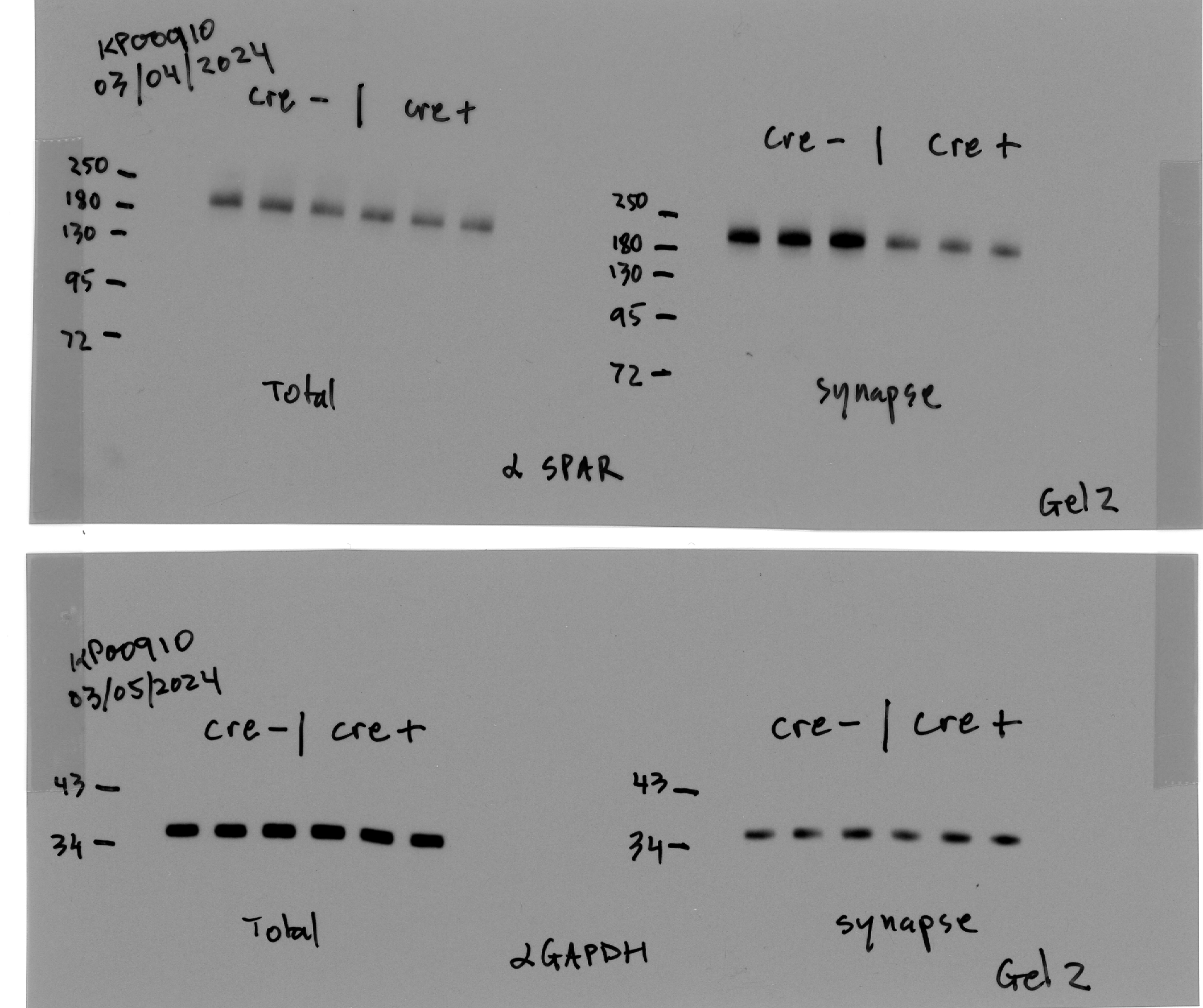

Supplement: Figure 2—source data 2. [file elife-100653-fig2-data2.zip › Figure 2 - Source Data 2/KP00910 gel2.tif]

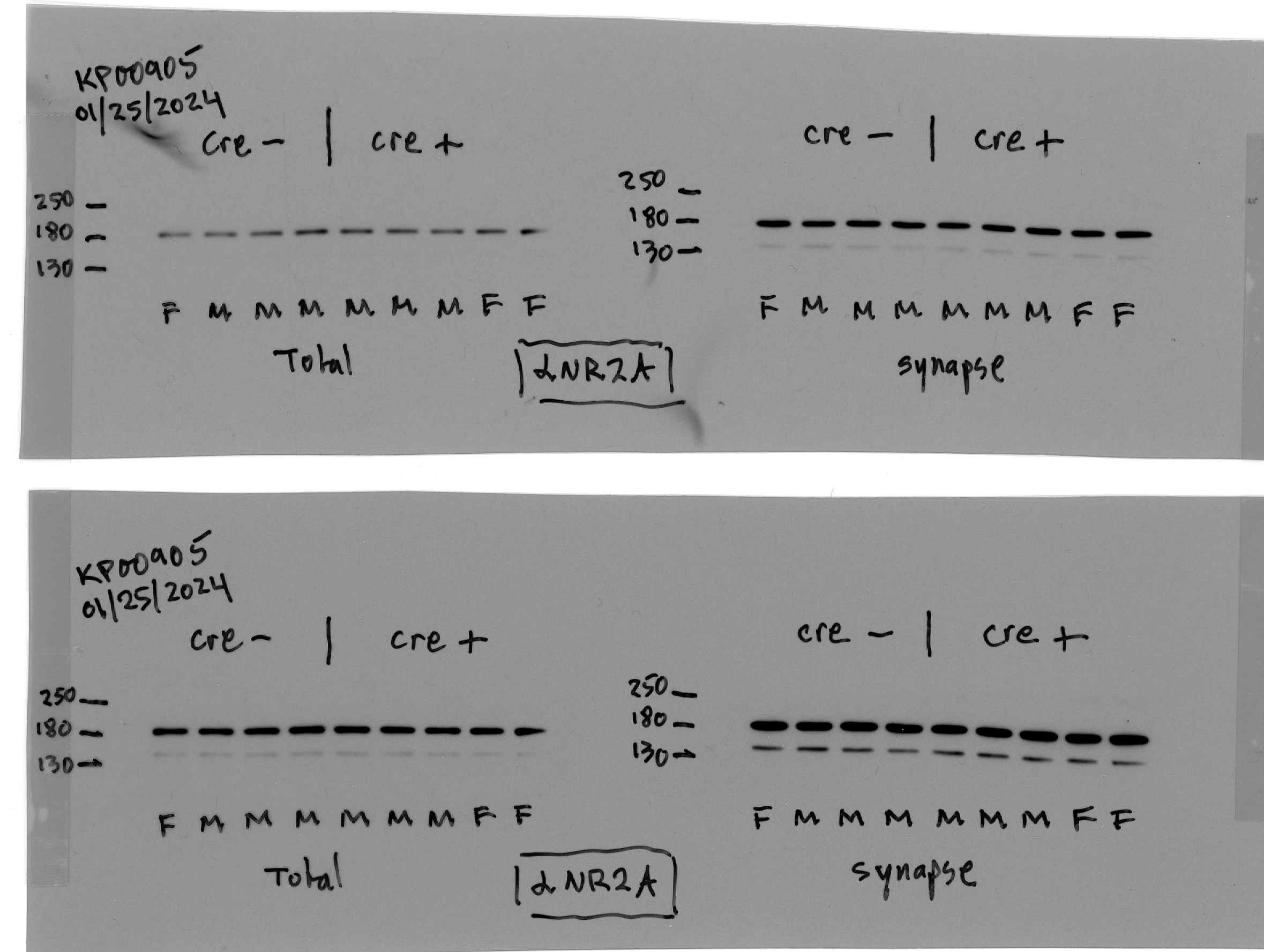

Supplement: Figure 2—source data 2. [file elife-100653-fig2-data2.zip › Figure 2 - Source Data 2/KP00905 NR2A.tif]

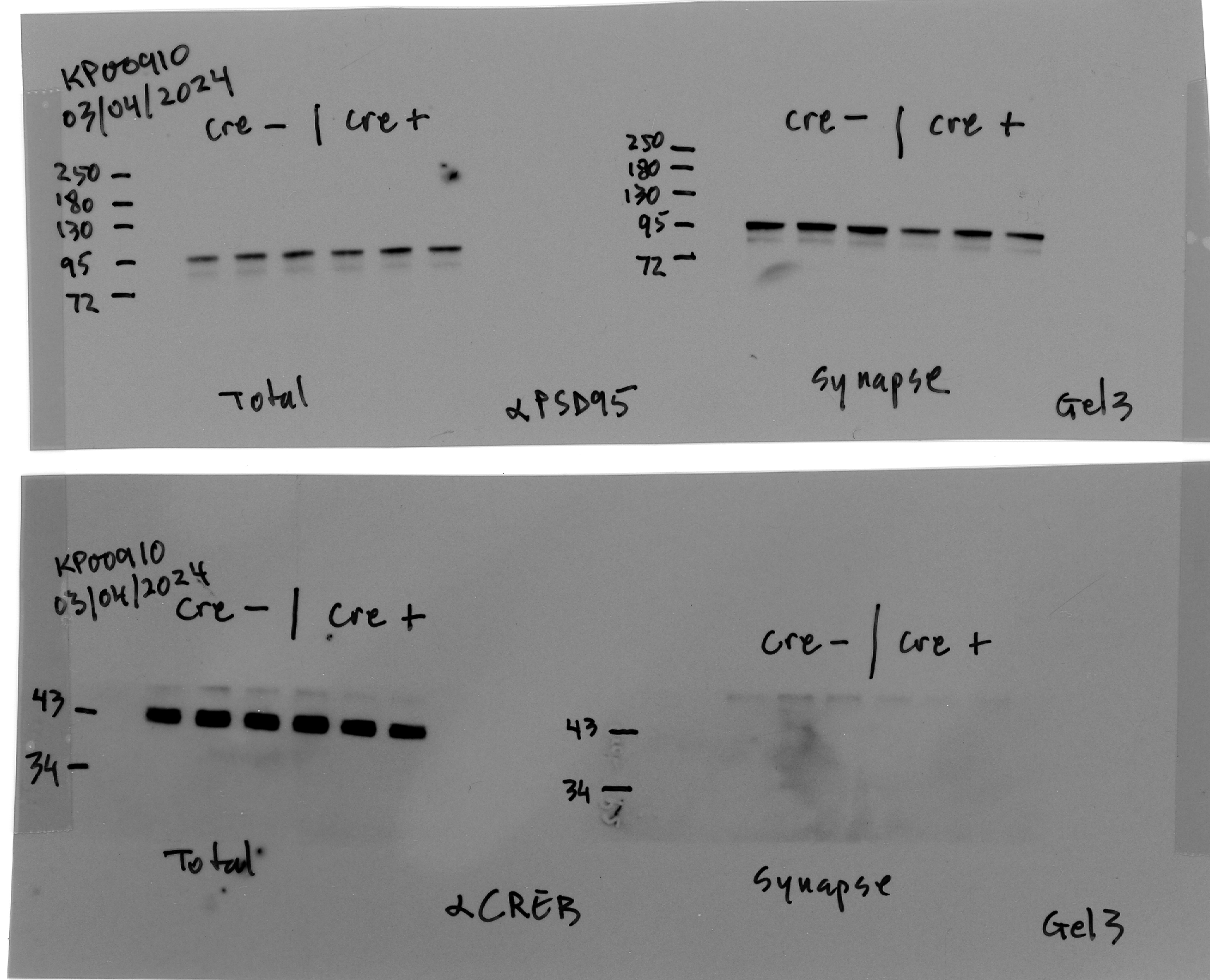

Supplement: Figure 2—source data 2. [file elife-100653-fig2-data2.zip › Figure 2 - Source Data 2/KP00910 gel3.tif]

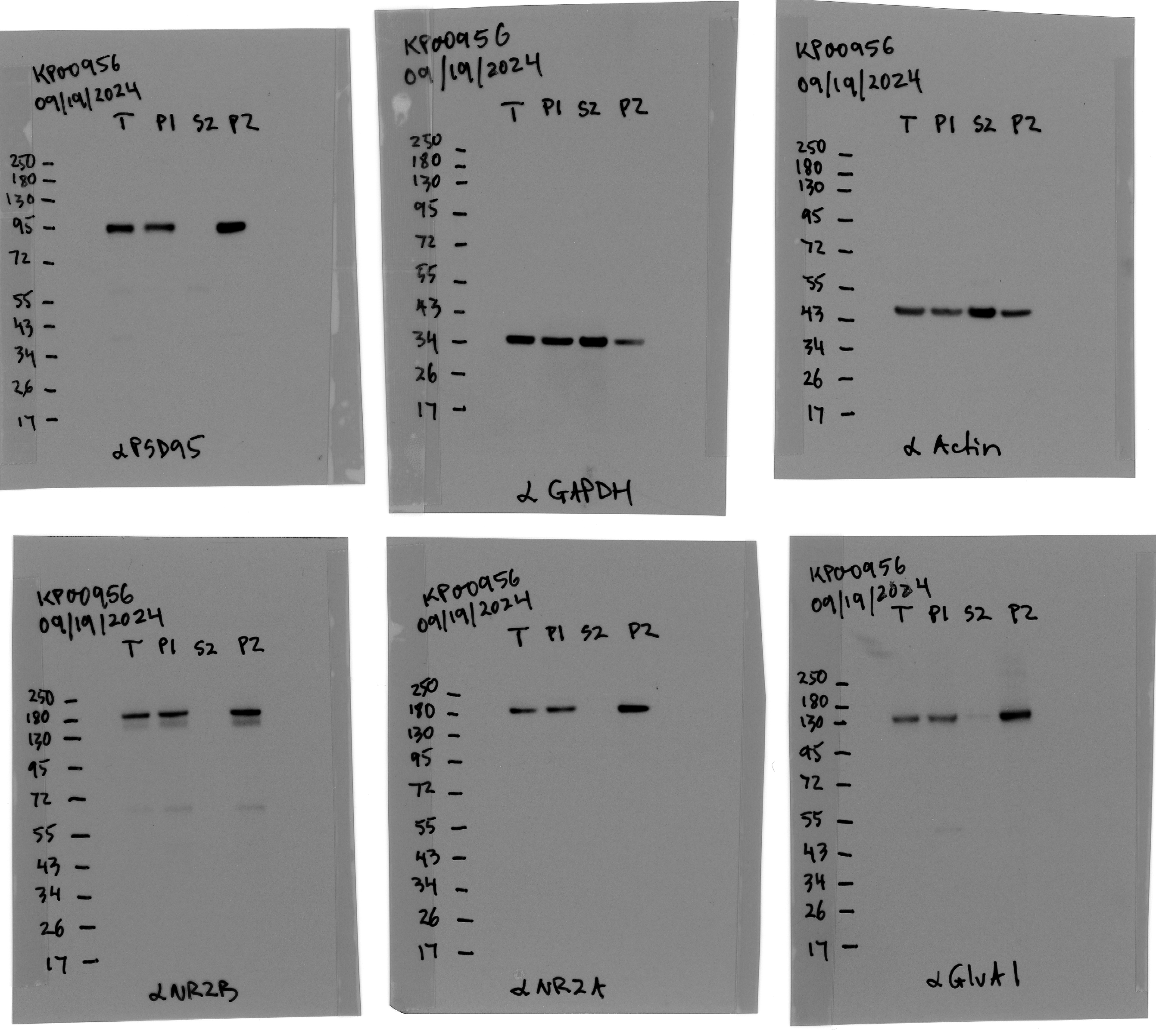

Supplement: Figure 2—figure supplement 1—source data 2. [file elife-100653-fig2-figsupp1-data2.zip › Figure 2 - Figure Supplement 1 - Data 2/KP00956.tif]

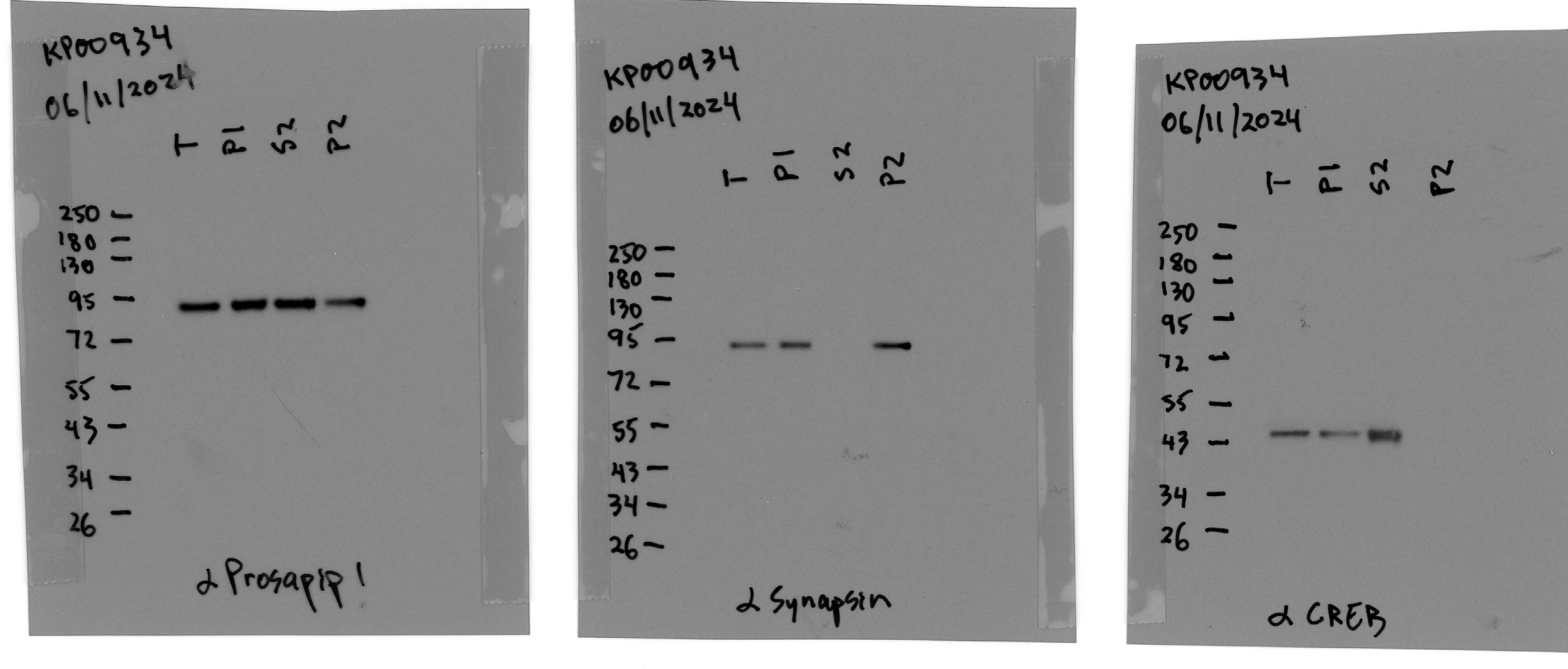

Supplement: Figure 2—figure supplement 1—source data 2. [file elife-100653-fig2-figsupp1-data2.zip › Figure 2 - Figure Supplement 1 - Data 2/KP00934.tif]

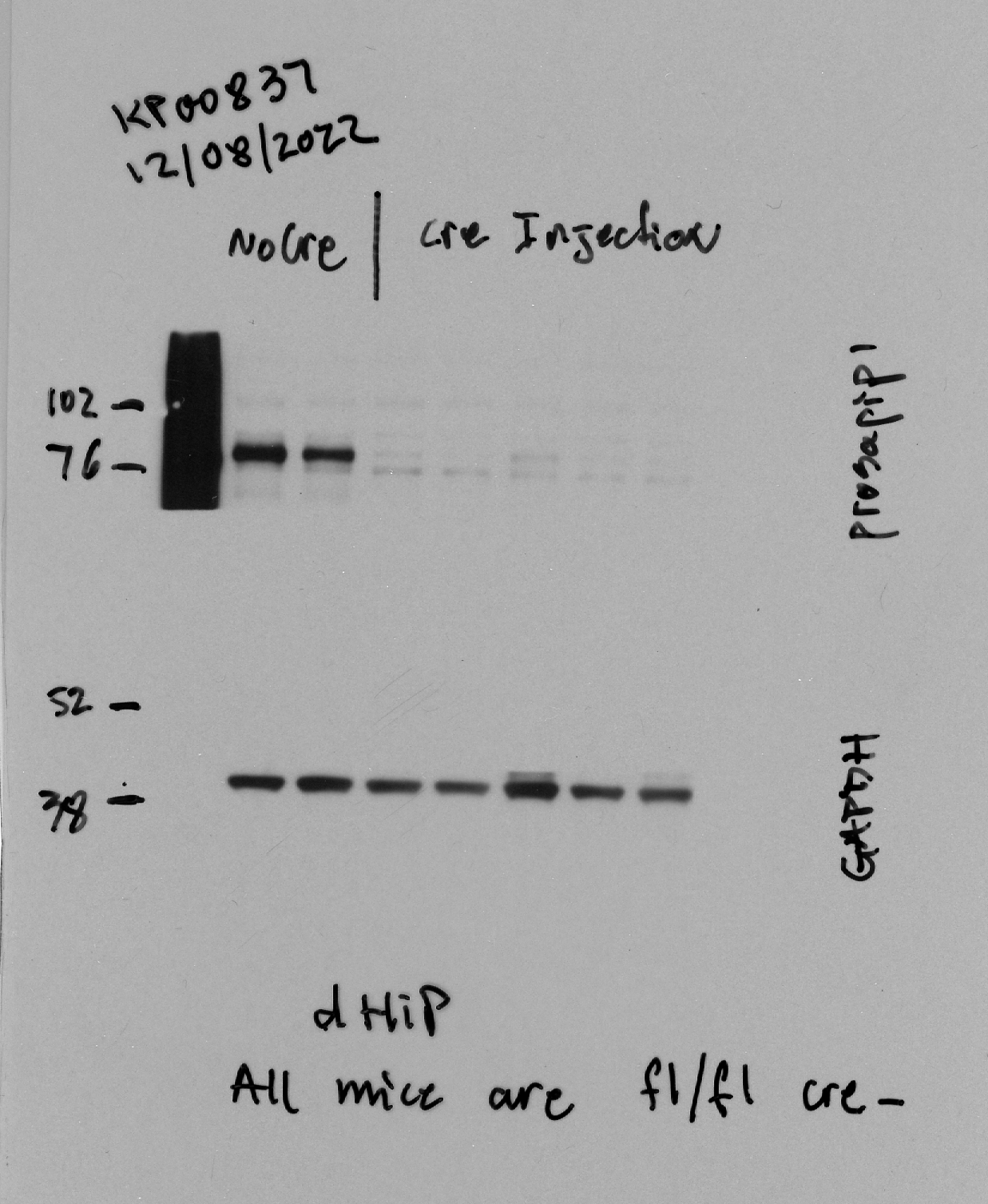

Supplement: Figure 5—source data 2. [file elife-100653-fig5-data2.zip › Figure 5 - Source Data 2/KP00837.tif]
